# Dysregulation of Oxygen Sensing/Response Pathways in Pregnancies Complicated by Idiopathic Intrauterine Growth Restriction and Early-Onset Preeclampsia

**DOI:** 10.3390/ijms23052772

**Published:** 2022-03-02

**Authors:** Sharon A. McCracken, Sean K. M. Seeho, Tamara Carrodus, Jenny H. Park, Narelle Woodland, Eileen D. M. Gallery, Jonathan M. Morris, Anthony W. Ashton

**Affiliations:** 1 Division of Perinatal Medicine, Faculty of Medicine and Health, The University of Sydney, Northern Sydney Local Health District Research (Kolling Institute), St. Leonards, NSW 2065, Australia; sean.seeho@sydney.edu.au (S.K.M.S.); tamara.corrodus@uts.edu.au (T.C.); jenny.seeho@yahoo.com.au (J.H.P.); eileeng@romtech.com.au (E.D.M.G.); jonathan.morris@sydney.edu.au (J.M.M.); anthony.ashton@sydney.edu.au (A.W.A.); 2 Department of Obstetrics and Gynaecology, Royal North Shore Hospital, St. Leonards, NSW 2065, Australia; 3 School of Biomedical Sciences, University of Technology Sydney, Ultimo, NSW 2007, Australia; nerelle.woodland@gmail.com

**Keywords:** placenta, pre-eclampsia, growth restriction, trophoblast, hypoxia, degradation, co-immunoprecipitation

## Abstract

Preeclampsia (PE) and intrauterine growth restriction (IUGR) are the leading causes of maternal and fetal morbidity/mortality. The central deficit in both conditions is impaired placentation due to poor trophoblast invasion, resulting in a hypoxic milieu in which oxidative stress contributes to the pathology. We examine the factors driving the hypoxic response in severely preterm PE (*n* = 19) and IUGR (*n* = 16) placentae compared to the spontaneous preterm (SPT) controls (*n* = 13) using immunoblotting, RT-PCR, immunohistochemistry, proximity ligation assays, and Co-IP. Both hypoxia-inducible factor (HIF)-1α and HIF-2α are increased at the protein level and functional in pathological placentae, as target genes prolyl hydroxylase domain (PHD)2, PHD3, and soluble fms-like tyrosine kinase-1 (sFlt-1) are increased. Accumulation of HIF-α-subunits occurs in the presence of accessory molecules required for their degradation (PHD1, PHD2, and PHD3 and the E3 ligase von Hippel–Lindau (VHL)), which were equally expressed or elevated in the placental lysates of PE and IUGR. However, complex formation between VHL and HIF-α-subunits is defective. This is associated with enhanced VHL/DJ1 complex formation in both PE and IUGR. In conclusion, we establish a significant mechanism driving the maladaptive responses to hypoxia in the placentae from severe PE and IUGR, which is central to the pathogenesis of both diseases.

## 1. Introduction

Preeclampsia (PE) and intrauterine growth restriction (IUGR) are common and potentially serious complications of pregnancy. Unexplained IUGR, the failure of the fetus to fulfil its growth potential, has no clinical impact on the mother but exposes the infant to long-term adverse health risks [1,2]. Early-onset PE, often accompanied by IUGR, is a multi-organ disorder characterised by maternal endothelial dysfunction, hypertension, and proteinuria [3]. Despite both conditions showing the constant pathological feature of abnormal placentation, the mother is only affected in PE. Moreover, PE may or may not be associated with impaired fetal growth.

Abnormal placentation arises from deficient spiral artery conversion, compromising utero-placental perfusion [4]. These vessels are at an increased risk of spontaneous vasoconstriction, contributing to the ischaemic injury of the placenta and the generation of oxidative stress [5]. Thus, placental expression of hypoxia-responsive genes (including hypoxia-inducible factor (HIF)-1α, HIF-2α) is increased in PE compared with normal controls [6,7,8,9]. Despite similarities in placental pathology, pregnancies complicated by late-onset IUGR do not show this hypoxic phenotype [10].

HIFs are heterodimers of α- and β-subunits belonging to the basic helix-loop-helix (bHLH)/Per-ARNT-Sim (PAS) domain family of transcription factors [11,12]. HIFs regulate the adaptive response of cells, tissues, and organisms to reduced oxygen availability [11]. Transcriptional responses to hypoxia are primarily mediated by HIF-1α and HIF-2α [13,14]. Under normoxia, the HIF-α-subunits are hydroxylated on key residues (P^402^ and P^564^) by prolyl hydroxylase domain (PHD) proteins 1–3. PHD1–3 require oxygen and function as oxygen sensors in vivo [15]. Prolyl hydroxylation of HIF-α-subunits recruits von Hippel–Lindau (VHL) protein that results in α-subunit ubiquitination and proteosomal degradation [16]. Under hypoxia, the inhibition of hydroxylation results in the dimerization of HIF-α-subunit with HIF-1β and enhanced transcription of the genes containing hypoxia-response elements in the promoter [17].

HIF-1α/-2α are indispensable for normal placental vascular morphogenesis [14] and trophoblast differentiation [18] in a variety of in vivo and in vitro systems; however, overexpression of HIF-α-subunits in women with PE is believed to be pathogenic [6,7,8,9]. The mechanism underlying the increased HIF-α expression in PE remains elusive; however, perturbation of oxygen-dependent degradation of HIF-α-subunits has been implicated [7,19]. Moreover, the dysregulation of HIF-α-subunit expression in IUGR is not well defined.

To determine whether the pathological mechanisms that underlie PE and idiopathic IUGR share a molecular signature, we compared the oxygen sensing/response pathways in the placentae from early-onset PE and severe IUGR with gestationally age-matched controls. We found HIF-1α and -2α, but not HIF-1β, expression was elevated in severe IUGR and early-onset PE, and disproportionately increased in the syncytiotrophoblast layer. The HIF-α-subunits were transcriptionally active as the level of target gene sFlt-1 was enhanced in both pathologies. This was despite elevated expression of PHD2 and 3 and factor inhibiting HIF (FIH) and no deficiency in Sprouty2 or VHL expression. In contrast, association of VHL and HIF-1α was impaired in pathological pregnancies, most likely due to the enhanced formation of complexes between VHL and its cognate inhibitor DJ1. Thus, a lack of substrate recognition by the ubiquitin ligase complex seems to be a central driver of the pathogenesis of these disorders.

## 2. Results

### 2.1. HIF-1α and HIF-2α Are Elevated in Early-Onset IUGR and PE Placentae

**Figure 1:**
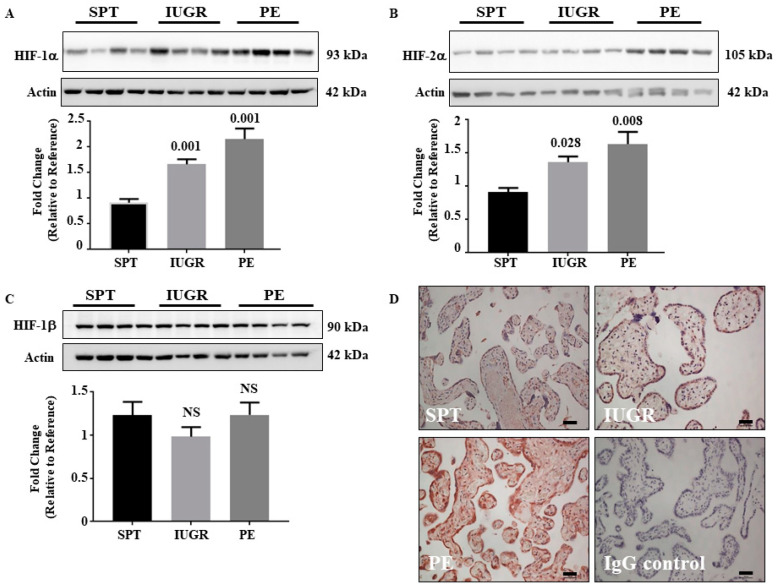
Expression of HIF subunits in placentae of normal and complicated pregnancies. (**A**–**C**) Representative Western blot and graph of HIF-1α (**A**), HIF-2α (**B**), and HIF-1β (**C**) in lysates from placentae of SPT (*n* = 13), IUGR (*n* = 16), and PE (*n* = 19) pregnancies. β-actin is included as a loading control. The densitometric value of one normally pregnant sample was normalised to one and changes observed in the other samples shown as a fold change compared to this reference sample. Representative blots and densitometric analysis of expression are shown. Data represent mean ± SEM values. *p* values as compared to normal pregnancy are indicated, NS = Not significant. (**D**) Immunohistochemical localisation of HIF-1α. Sections of placentae from Normal (*n* = 13), IUGR (*n* = 16), and PE (*n* = 19) pregnancies were immunostained for the presence of HIF-1α. Negative control was performed using non-immune mouse IgG antibody. Images are representative of each group. Scale bare represents 100 μm (original magnification, ×100). Full gels of immunoblots can be found in SF1.

**Figure 2:**
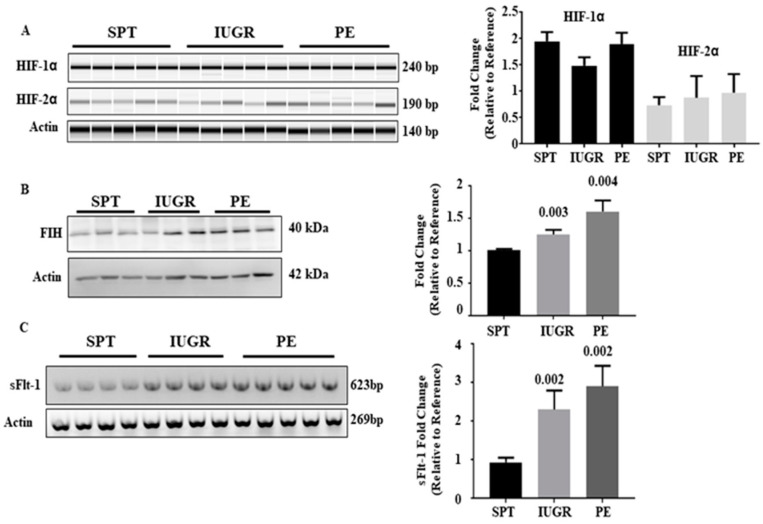
HIF gene regulation, expression of Factor inhibiting HIF (FIH) and Expression of the HIF regulated gene sFlt-1 in placental tissues. (**A**) mRNA of normal (*n* = 13), IUGR (*n* = 16), and PE (*n* = 19) pregnancies was probed for HIF-1α and 2α transcript levels by RT-PCR. (**B**) Placental lysates from normal (*n* = 13), IUGR (*n* = 16), and PE (*n* = 19) pregnancies were probed for FIH expression. Immunoblotting for β-actin was used to control for loading. (**C**) mRNA of normal (*n* = 13), IUGR (*n* = 16), and PE (*n* = 19) pregnancies was probed for sFlt-1 transcript levels by RT-PCR. β-actin transcript mRNA levels were assessed as a control (**A** and **C**). The densitometric value of one normal pregnancy control was normalised to one and changes observed in the other samples shown as a fold change compared to this reference sample. Representative blots/gels and densitometric analysis of expression are shown. Data represent mean ±SEM. *p* values as compared to normal pregnancy are indicated. NS = Not significant. Full gels for immunoblots can be found in SF2.

Abnormal placentation is common in both IUGR and PE. Although oxygen handling is perturbed in PE, it has not been well investigated in IUGR. Figure 1 shows that placental HIF-1α levels (Figure 1A) were 1.8 (*p* = 0.001) and 2.4 (*p* = 0.001)-fold higher in IUGR and PE samples relative to SPT controls, respectively. Similarly, the levels of placental HIF-2α (Figure 1B) were 1.5 (*p* = 0.028)- and 1.8 (*p* = 0.008)-fold greater in IUGR and PE, respectively. There was no significant difference (*p* > 0.05) in either HIF-1α or HIF-2α levels between PE and IUGR samples. HIF-α-subunits dimerise with HIF-1β to alter transcriptional responses. Unlike HIF-α-subunits, HIF-1β is constitutively expressed [20], and protein levels were comparable between all groups (Figure 1C). The changes in HIF-1α and 2α expression were not reflected by altered transcription as the mRNA levels were identical between groups (Figure 2A).

### 2.2. HIF-1α Expression Is Ubiquitous in Placentae Affected by IUGR and PE

Having demonstrated increased HIF-α-subunit levels in placentae from pathological pregnancies, we examined the location of HIF-1α (Figure 1D). The immunostaining confirmed Western blot data showing HIF-1α expression in SPT controls with an enhanced expression in placentae of IUGR and PE relative to SPT age-matched controls (Figure 1D). Moreover, staining intensity was greater in PE than in IUGR placentae (Figure 1D). Although these data indicate that the increase in HIF-α-subunit expression was not confined to a particular cell type, the syncytiotrophoblast layer exhibited a disproportionate increase in staining compared to other cell types.

### 2.3. Increased HIF Expression Translates to Altered Expression of sFlt-1 mRNA in IUGR and PE Placentae

We next determined whether the increase in HIF expression was functional. FIH controls transcriptional activity of HIF-α-subunits under hypoxic conditions by inhibiting the association with p300/CBP [15,17]. Expression of FIH (Figure 2B) was 1.3- and 1.6-fold elevated in placental lysates affected by IUGR (*p* = 0.003) and PE (*p* = 0.004), respectively, compared with SPT. As the increase in FIH expression mirrored that in HIF, we examined soluble (s)Flt-1 expression as a direct transcriptional target of HIF-1α [21,22]. Placental levels of mRNA for sFlt-1 are increased in IUGR and PE pregnancies by RT-PCR, by 2.2- and 3.1-fold, respectively, compared with SPT controls (Figure 2C) (*p* < 0.05). Thus, the upregulated HIF-1/2α was transcriptionally active despite increased FIH and may contribute significantly to the pathology of these conditions through up-regulating the molecular drivers of abnormal pregnancies, especially in PE.

### 2.4. The HIF-1/2α Regulatory Apparatus Is Not Absent in PE and IUGR

**Figure 3:**
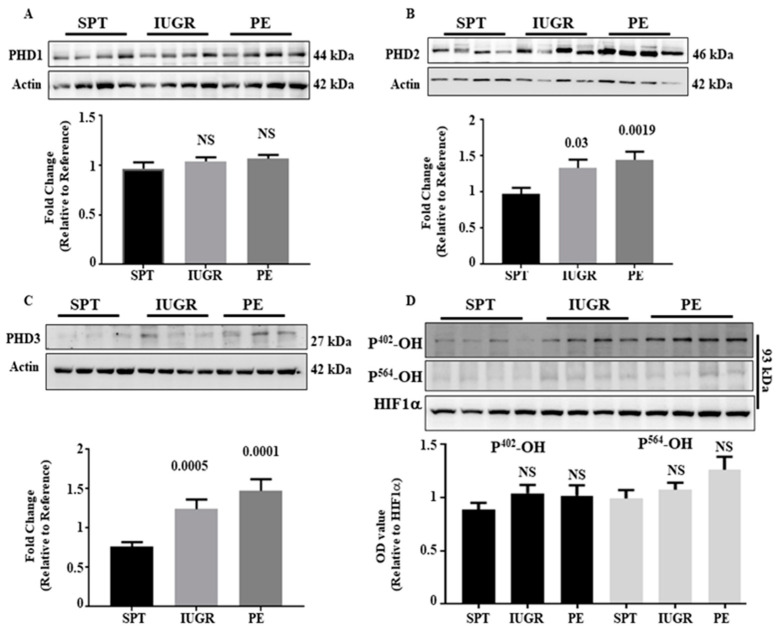
Expression of HIF-1/2α regulatory proteins in the placentae of complicated pregnancies. Expression of the HIF-α regulatory proteins PHD1 (**A**), PHD2 (**B**), and PHD3 (**C**) was assessed by immunoblotting in placentae of normal (*n* = 13), IUGR (*n* = 16) and PE (*n* = 19) pregnancies. Representative Western blot and densitometric analysis of expression are shown. β-actin is included as a loading control. (**D**) Function of PHD enzymes was assessed by immunoblotting for hydroxylated P^402^ and P^564^ in HIF-1α. In this case immunoblotting for HIF-1α was used as the loading control. The value of one normal pregnancy control was normalised to one and changes observed in the other samples shown as a fold change compared to this reference sample. Data represent mean ± SEM. *p* values as compared to normal pregnancy are indicated. NS = Not significant. Full gels for immunoblots can be found in SF3 and SF4.

Figure 1 and Figure 2 suggest that HIF-α-subunit upregulation was post-transcriptionally regulated. When oxygen is available, HIF-α-subunits are hydroxylated at either Pro^402^, Pro^564^, or both, by PHD proteins 1–3 driving association with VHL and proteosomal degradation [15,17,23,24]. Analysis of placental lysates demonstrated no significant difference in PHD1 expression between the three groups (Figure 3A); however, PHD2 levels were increased 1.4- and 1.5-fold in the IUGR (*p* < 0.05) and PE (*p* < 0.01) groups, respectively, compared with SPT (Figure 3B). Similarly, PHD3 expression was augmented 1.3 (*p* < 0.01)- and 1.5 (*p* < 0.01)-fold relative to SPT (Figure 3C). However, no differences in PHD2 and PHD3 expression were observed between IUGR and PE (*p* > 0.05). The enhanced expression of PHD2 and PHD3 in IUGR and PE is consistent with the elevated HIF-1/2α being transcriptionally active (Figure 2), as both are HIF targets [25]. Thus, HIF-1/2α accumulation in diseased placentae is not associated with deficient PHD expression. We assessed PHD enzymatic activity by determining the hydroxylation status of HIF-α-subunits using an antibody specific to hydroxylated-P^402^ and P^564^. As HIF-1α expression was higher in placental lysates from IUGR and PE pregnancies (Figure 1), non-hydroxylated HIF-1α was used as a control. Hydroxylation at residues 402 (HIF-P^402^) and 564 (HIF-P^564^) was not significantly different between groups (Figure 3D), suggesting substrate engagement and modification by the PHDs was similar in normal and pathological pregnancies and enhanced expression was not due to failed initiation of the degradation process in IUGR and PE.

**Figure 4:**
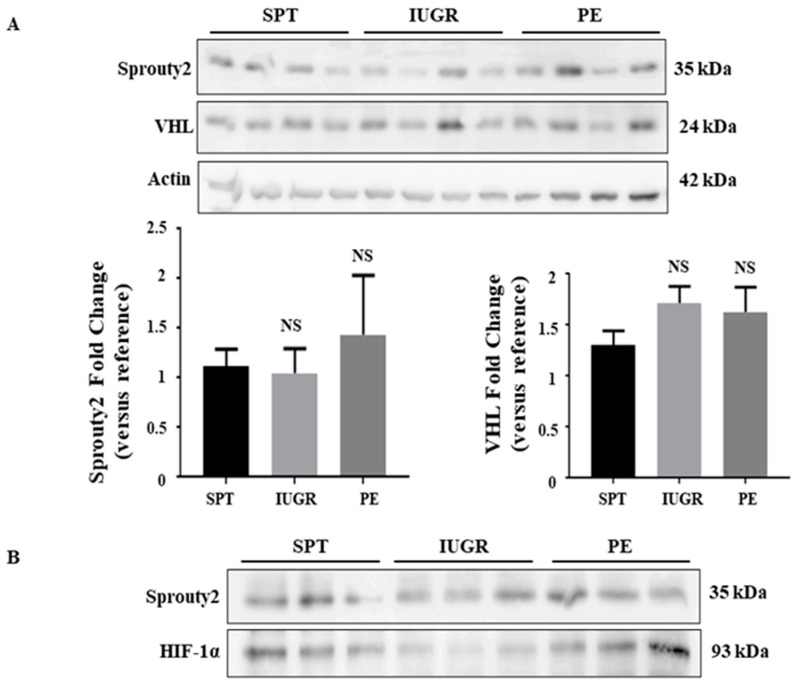
Expression of VHL and Sprouty2 in the placentae of complicated pregnancies. (**A**) Expression of the HIF-α regulatory protein VHL and Sprouty2 was assessed in placentae of normal (*n* = 13), IUGR (*n* = 16), and PE (*n* = 19) pregnancies by immunoblotting. Representative Western blot and densitometric analysis of expression are shown. β-actin is included as a loading control. The value of one normal pregnancy control was normalised to one and changes observed in the other samples shown as a fold change compared to this reference sample. Data represent mean ± SEM. NS = not significant as compared to normal pregnancy. (**B**) Detection of Sprouty2 by immunoprecipitation in SPT (*n* = 3), IUGR (*n* = 3), and PE (*n* = 3) tissues. Protein complexes were identified using anti-HIF-1α and Srpouty2. HIF-1 α was used as the pull-down antibody. Full gels for immunoblots and Co-IP can be found in SF5.

Under normoxic conditions, hydroxylated HIF-α is bound by VHL protein, which targets it for degradation by the 26*S* proteasome [20]. Sprouty2, the scaffold on which this complex is formed, is also essential for both HIF-1α and -2α degradation [26]. A deficiency of either Sprouty2 or VHL protein in IUGR and PE pregnancies would therefore increase HIF-1α/2α in these conditions. The immunoblotting of placental lysates demonstrated no significant difference in the expression of either Sprouty2 or VHL in diseased versus normal tissues (Figure 4A). Moreover, immunoprecipitation analysis revealed that normal or enhanced complex formation between Sprouty2 and HIF-1α existed in IUGR and PE tissues (Figure 4B). Thus, the accumulation of HIF-1α/2α in diseased placentae is not due to any overall deficiency in the expression or function of any regulatory protein.

### 2.5. The Interaction of HIF-1α and VHL Is Greatly Diminished in PE and IUGR

**Figure 5:**
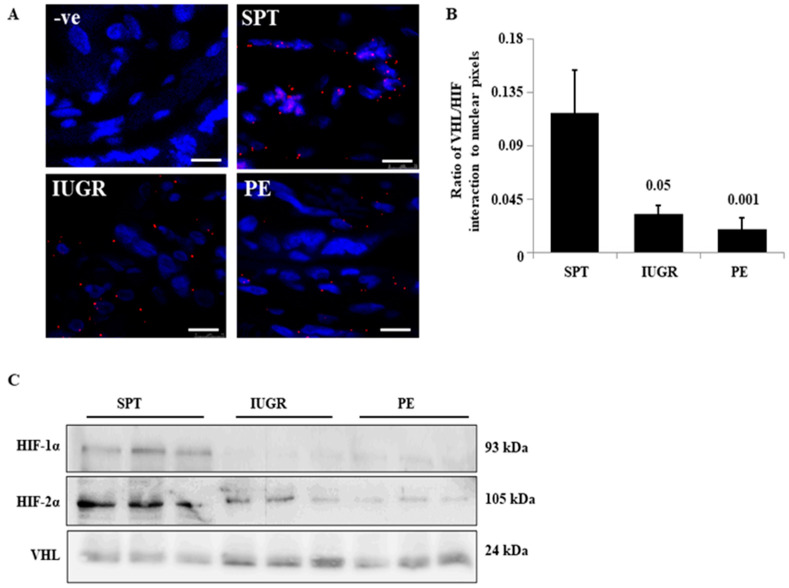
Interaction of HIF-1α and VHL in the placentae of complicated pregnancies in situ. (**A**) The interaction of HIF-1α and VHL was assessed in situ for normal (*n* = 5), IUGR (*n* = 4), and PE (*n* = 4) pregnancies using the proximity ligation assay. The representative images show the interaction as red dots. (**B**) Staining was quantified by normalising signal from the PLA (pixel counts) with nuclear staining from the DAPI counterstain. *p*-values are compared to normal pregnancy. Scale bare represents 100 μm (original magnification, x100) (**C**) Complex formation was assessed in lysates of normal (*n* = 3), IUGR (*n* = 3), and PE (*n* = 3) by immunoprecipitation, pull-down was performed using anti-VHL and complexed HIF-1α and 2α were assessed by immunoblotting. Full gels for Co-IP can be found in SF6.

Despite intact oxygen sensing in diseased placental tissue, there is persistent HIF-α-subunit dysregulation. We therefore tested whether post-translationally modified HIF α-subunit interactions were altered. HIF-1/2α interacts with VHL prior to entry into the proteasome. Using the proximity ligation assay (PLA), HIF-1α/VHL complex formation (red dots) was observed in SPT placentae (Figure 5A). The number of HIF-1α/VHL complexes per cell was greatly reduced in IUGR (72.1 ± 17.9% reduction; *p* ≤ 0.05) and further reduced in PE (84.3 ± 25.4% reduction; *p* ≤ 0.01) (Figure 5A,B). Consistent with the PLA, immunoblotting of VHL containing protein complexes from co-immunoprecipitation demonstrated HIF-1α and -2α complexed with VHL in all SPT samples tested but was reduced in all IUGR and PE samples (Figure 5C). Thus, the deficit in the HIF-1α regulation in PE and IUGR appears to result from altered VHL and HIF-α subunit interactions.

**Figure 6:**
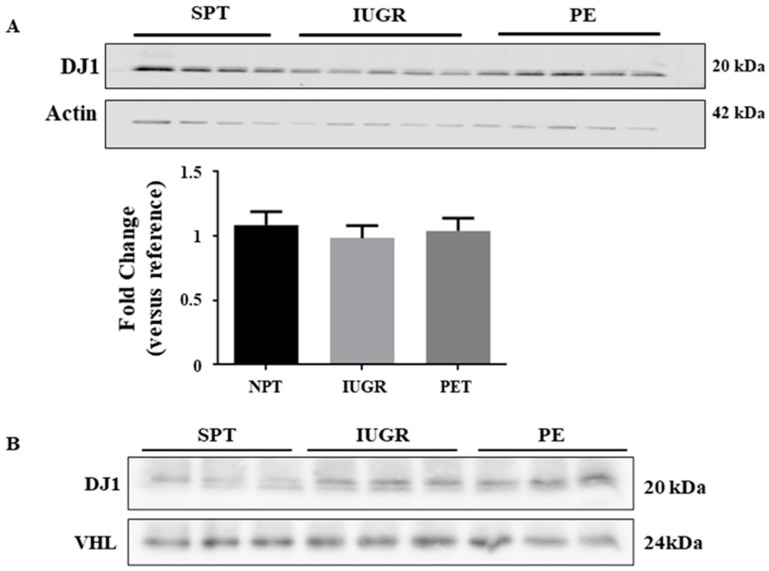
Expression of PARK7/DJ1 in the placentae of complicated pregnancies. (A)Expression of the VHL binding protein PARK7/DJ1 was assessed in placentae of normal (*n* = 6), IUGR (*n* = 7), and preeclamptic (*n* = 11) pregnancies by immunoblotting. Representative Western blot and densitometric analysis of expression are shown. β-actin is included as a loading control. The value of one normal pregnancy control was normalised to one and changes observed in the other samples shown as a fold change compared to this reference sample. Data represent median values ± SEM. (**B**) Complex formation was assessed in lysates of normal (*n* = 3), IUGR (*n* = 3), and PE (*n* = 3) by immunoprecipitation, pull-down was performed using anti-VHL and complexed DJ1 were assessed by immunoblotting. Full gels for immunoblots and Co-IP can be found in SF7.

A lack of VHL/HIF-1α interactions may be explained by competitive inhibition of the complex by a third protein. DJ1 (a competitive binding protein of VHL/HIF-α interactions) [26,27] has been identified in the placenta, with increased expression in the syncytiotrophoblast layer of PE placentae [28]. Contrary to the previous report, we found no significant difference in DJ1 expression between normal and either PE or IUGR affected placentae (Figure 6A), nor was there a difference in DJ1 distribution (data not shown). Co-immunoprecipitation indicated low levels of DJ1/VHL complexes were observed in SPT samples; however, these complexes were greatly enhanced in IUGR and PE samples (Figure 6B). Thus, the failure of VHL/HIF-α complex formation in pathological pregnancies was likely a direct result of competitive binding by DJ1.

## 3. Discussion

The present study demonstrates that HIF-1α and HIF-2α protein expression is elevated at the post-transcriptional level in the placentae of normotensive women with severe IUGR and women with early-onset PE. As previously reported, HIF-1α/2α is transcriptionally active [6] with enhanced expression of target genes (*sFlt-1*, PHD2, and 3). Accumulation of HIF-α-subunits in our patient cohort was not due to deficient proline hydroxylation (P^402^), expression of PHD1, 2, or 3, or VHL proteins (which initiate the degradation of HIF-α subunits). However, abrogated HIF-1α/VHL complex formation was observed resulting from aberrant formation of VHL-DJ1 complexes. The observed accumulation of HIF-1α/2α in PE samples is consistent with findings from earlier studies [6,8,9,10]. Notably, although HIF-α subunits are temporally regulated throughout gestation [29], they are clearly detectable in normal term placental tissues when the placenta is sufficiently oxygenated, which also supports previous reports [30,31].

The data on HIF-α-subunit regulation in IUGR is conflicting. We show increases in HIF-α subunits in severe early-onset IUGR (delivered at <34 weeks with birthweight < third centile), which may reflect a more severe placental pathology compared with late-onset IUGR pregnancies (≥37 weeks of gestation, asymmetric growth, and birth weight < 10th centile). Previous studies assessing IUGR placental tissue with gestations > 36 weeks have shown similar [10], increased [30], or reduced [32] levels of HIF α-subunits. The discrepancies between each study have largely been attributed to the severity of the IUGR cohort examined, with those showing a more severe phenotype largely showing an increased HIF-α-subunit expression [30]. Our data showing increased HIF-1α/2α expression in early-onset IUGR is consistent with the recognition that IUGR placentae show reduced morphological changes of spiral arteries in placental bed and that the lack of alterations is more pronounced in more severely growth restricted fetuses [33]. The lack of spiral artery remodelling results in compromised placental perfusion and subsequent oxygen/nutrient delivery, ultimately resulting in abrogation of oxygen regulation. Additionally, it is increasingly apparent that early-onset IUGR and PE have distinct molecular phenotypes compared to their late-onset counterparts [19] In fact, the reduced placental perfusion characteristic of early-onset PE represents a different subtype of disease than PE at term when placental-malperfusion may not be as relevant [34]. Whilst our study concurs with previous studies on early-onset PE, our findings may not be relevant to late-onset disease [35]. The current study focused on severe early-onset IUGR and early-onset PE. Our data demonstrate similarities in the molecular pathology of early-onset PE and severe early-onset IUGR, although the degree of HIF-α subunit stabilisation is greater in PE. The more pronounced alterations in PE may reflect greater impairment of spiral artery remodelling compared with IUGR pregnancies as previously documented [34].

The failure to effectively degrade HIF-1α in the placentae of PE or IUGR pregnancies is thought to be the main driver of disease [7]. Possible mechanisms for the stabilisation of HIF-1α levels in early-onset PE and IUGR could be failure to respond to tissue oxygenation or failure to degrade the protein. Our data suggest that oxygen sensing is intact, and the deficit lies in the degradation pathway. However, a previous study of early PE tissue showed decreased PHD2 expression and HIF-1α proline hydroxylation associated with accumulation of HIF-1α suggesting impaired oxygen sensing [19]. Although our results do not validate these earlier findings, we do show that HIF-α stabilisation in our cohort is associated with a failure for VHL to bind HIF-α-subunits, limiting targeted degradation. It is possible that both mechanisms exist and may reflect differences in subclasses of PE, since it has been shown that PE has been classified into four distinct subclasses based on microarray analysis [36] and neither cohort has been phenotyped.

In our cohorts, accumulation of HIF-α-subunits in IUGR and PE placentae occurred despite normal expression of PHD1–3 and VHL, confirming a previous report with similar tissue [37]. We have now determined that hydroxylation of P^402^ and P^564^ in HIF-1α is also normal, suggesting PHD2 and 3 were active [20,38]. The upregulation of PHD2 and 3 in the placentae of severe IUGR and PE pregnancies parallels the increases in HIF-α proteins in these conditions. Both PHD2 and 3 are themselves HIF targets, and their mRNAs are accordingly induced by hypoxia [17]. Moreover, Sprouty2, the scaffold that enables VHL recruitment to hydroxylated HIF-α increasing ubiquitination and degradation [26], was similarly intact. Overall, our data suggests there were no deficiencies in the molecular machinery required for appropriate oxygen regulation.

The primary deficit we show here is a failure of VHL and HIF-1α to interact, which we have shown by both proximity ligation assays and immunoprecipitation. This observation is not supported by an earlier report [39], which used co-immunoprecipitation to confirm VHL-HIF-1α interactions were intact in placentae from PE pregnancies. However, Rajakumar et al., compared PE samples (35.9 ± 0.8 weeks) with term controls (39.8 ± 0.3 weeks). It is possible that the differences in gestational ages account for the discrepancies between our two studies. Alternatively, this may similarly reflect different PE phenotypes.

Post-translational modification of HIF-1α and the new binding partners for VHL could explain our findings. Multiple proteins interact with VHL [16,40], but few have been validated for their effects on HIF-α-subunit degradation, and fewer still [41] are characterised as being perturbed in pathological pregnancies. The VHL binding protein DJ1 regulates the function of HIF-1α in both cancer and neuronal cells through binding VHL and limiting HIF-1α degradation [27,28]. DJ1 was reported to be increased in the placentae of PE women [42,43]. However, although we detected DJ1 in placental tissue from our PE and IUGR cohorts, there was no evidence of increased expression relative to controls. While we compared levels of DJ1 in early gestation samples from normal and PE pregnancies, Yang et al. [43] compared DJ1 expression in early-onset preeclamptic placentae with late control placental tissues. The use of SPT controls in our study and term tissues by Yang and colleagues may explain these differences as it is currently unknown whether expression of DJ1 varies with gestational age similar to HIF-1α. In contrast, we showed that binding of DJ1 to VHL increased in both PE and IUGR, suggesting a significant new mechanism of HIF-1α stabilisation in early-onset PE and IUGR. Moreover, DJ1 deficiency leads to reduced HIF-1α levels in models of both hypoxia and oxidative stress [28], which highlights the possibility that this mechanism plays a significant role in these pregnancy pathologies. To our knowledge, this is the first-time competition for VHL binding between DJ1 and HIF-1α/2α has been identified as a potential mechanism for stabilising HIF-1α in either severe PE or IUGR. Further research is required to fully understand these interactions and determine whether, clinically, this interaction is a potential target for the development of a therapy for PE and or IUGR.

A key question is whether the accumulation of HIF-α-subunits in the placentae of PE and IUGR is the cause of disease or a maladaptation to the pathological stress in the developing placenta. Using adenoviral delivery, HIF-1α overexpression in pregnant mice (but not non-pregnant mice) increased blood pressure, retarded fetal and placental growth, and promoted glomerular endotheliosis and proteinuria [44]. As such, the up-regulation of HIF-α-subunits would appear to be causal in the pathogenesis of both PE and IUGR.

The question remains as to why some women develop IUGR and others develop PE when the placental molecular changes are similar in both conditions, although more so in PE. It may be that in some women, the placental changes and release of factors from the stressed placenta (such as sFlt-1, sEng, and syncytiotrophoblast membrane microparticles) are sufficient to cause the maternal syndrome of PE. Alternatively, maternal factors may play a role in affecting the susceptibility of women to developing PE. Indeed, Ness and Sibai 2006 [45] postulated that the presence of maternal metabolic syndrome triggers the development of PE in conjunction with abnormal placentation and that IUGR develops in the absence of antenatal metabolic syndrome. Maternal constitutional factors may also influence whether the maternal syndrome of PE develops [46]. It is therefore likely that since PE is a heterogeneous disorder and not all factors proposed to cause the disorder are found in all women with the condition, that both maternal and placental factors may contribute to varying degrees in individual cases or distinct phenotypes. Although sufficient pathological placentae were utilised to assess HIF regulation, a limitation of our study is that the placental tissue was not phenotyped, thus distinct sub-groups were not identified.

In conclusion, we have shown that persistent HIF-1α/2α in the placenta of early-onset IUGR and PE pregnancies is due to a lack of VHL binding and this is determined by the level of binding of the HIF competitor DJ1. However, given the heterogenicity associated with pathological placentation, it is unlikely that one underlying mechanism drives all IUGR and/or PE. Rather, there are likely multiple mechanisms that exist that may be specific to the phenotype associated with the pathology.

## 4. Materials and Methods

### 4.1. Subjects

Ethics approval for the study was obtained from the Northern Sydney Local Health District Human Research Ethics Committee (approval no. 0912–348M). Written informed consent was gained from all subjects before they participated in the study in accordance with the Declaration of Helsinki. Placentae were obtained after caesarean section from women with spontaneous pre-term pregnancy (SPT, *n* = 13), IUGR (*n* = 16) and PE (*n* = 19). All SPTs presented with pre-term premature rupture of membranes (PPROM). One was delivered vaginally and the rest by caesarean section (CS) for previous CS, vasa previa, breech presentation, or oligohydramnios. Table 1 summarises clinical data. PE was diagnosed as per Australasian (ASSHP) and International (ISSHP) guidelines ([47,48] as de novo hypertension (systolic pressure ≥ 140 and/or a diastolic pressure ≥ 90 mmHg) and spot urine protein/creatinine ratio ≥ 30 mg/mmol occurring >20 weeks gestation)). IUGR was defined as birth weight < 3rd percentile for gestational age/sex [49], and antenatal evidence of abnormal umbilical artery Doppler flow. PE and IUGR were classified as severe based on the gestational age at delivery (≤34 weeks). Other than PPROM all SPTs were devoid of clinically significant conditions.

### 4.2. Tissue Collection

Tissues were sampled immediately following delivery. Areas of macroscopic calcification, infarction and haemorrhage were avoided. Tissues were dissected, rinsed in sterile cold PBS, blotted dry and snap frozen in liquid nitrogen or stored in RNAlater™ (Qiagen, Hilden, Germany). Frozen and RNAlater samples were stored at −196 °C and −80 °C, respectively. A separate tissue biopsy was taken for histological analysis. Tissues were fixed in 10% (*v/v*) neutral buffered formalin overnight and processed for paraffin-embedding.

### 4.3. Immunoblot

Protein was extracted from villous tissues using the PARIS™ isolation kit with Protease Inhibitor Cocktail Set III (Calbiochem, San Diego, CA, USA) according to manufacturer’s instructions (Ambion, Sydney, NSW, Australia). Protein concentrations were measured (Bradford protein assay; Bio-Rad, Hercules, CA, USA), 50 μg separated on NuPAGE^®^ Novex^®^ mini gels (Invitrogen, Waltham, MA, USA), transferred to PVDF membranes and immunoblotting performed as described [50], using primary antibodies against HIF-1α (1:1000, Abcam, Cambridge, UK), HIF-2α (1:1000), PHD1–3 (1:500) (Novus Biologicals, Littleton, CO, USA), Sprouty2 (1:1000; Santa Cruz, Dallas, TX, USA), HIF-1β (1:500), VHL (1:1000), DJ1 (1:1000), and the hydroxylated form of HIF-1α (P^402^-OH (1:500) and P^564^-OH (1:500) (Abcam, Cambridge, UK). HIF-1α served as a loading control for the hydroxylated proline blots and β-actin (1:3000 Sigma, Burlington, MA, USA) for the rest. Bound antibody was detected using the appropriate HRP conjugated antibody and chemiluminescence (PerkinElmer, Waltham, MA, USA). Images were captured using the LAS 4000 imaging system (Fujifilm, Macquarie Park, NSW, Australia). Densitometry analysis was performed using the Image-J digital software version 1.8.0 with bands of interest standardised against actin or HIF as indicated.

### 4.4. Co-Immunoprecipitation

Isolation of HIF-1α and Sprouty2 as well as VHL, HIF-1α, or DJ1 containing protein complexes was performed as previously described [51] using 4 µg of pull-down antibody and 400 mg of total protein lysate. To equalise the number of HIF complexes 1000 mg of SPT proteins was used for HIF-1α isolation. SDS-PAGE, electro transfer, and immunoblotting were performed as described above.

### 4.5. RNA Isolation and Semi-Quantitative RT-PCR

Total RNA was isolated from placental tissue in RNAlater using the RNeasy^®^ Mini Kit (Qiagen, Hilden, Germany) and RNA concentrations determined using the Nanodrop. RNA (1 μg) was reverse transcribed using SuperScript™III (Invitrogen, Waltham, MA, USA). PCR primers and conditions are outlined in Table 2. RT-PCR products were separated by electrophoresis on 1% (*w*/*v*) agarose gels or a microchip electrophoresis system (MultiNA; Shimadzu, Kyoto, Japan) as indicated. Relative intensities of bands for HIF-1α, HIF-2α and sFlt-1 were quantitated after normalisation to β-actin using MultiGauge version 3.1 software (Fujifilm, Macquarie Park, NSW, Australia).

### 4.6. Immunohistochemistry (IHC)

Sections (4 μm) of paraffin-embedded placental tissue were stained as described [52] with antigen retrieval at pH6.0 for 20 min. Sections were incubated with antibodies against HIF-1α (Abcam, Cambridge, UK) at 2 μg/mL over night at 4 °C. IHC staining was detected using the ENVISION™ system (DAKO, Santa Clara, CA, USA) with NovaRED™ (Vector Labs, Burlingame, CA, USA) as the substrate. Tissue sections were counterstained with Harris haematoxylin. Images were captured using NIS Element software (Nikon, Tokyo, Japan) via Nikon Digital Sight (H550S, Nikon, Tokyo, Japan).

### 4.7. In Situ Proximity Ligation Assay (PLA)

PLA was performed on tissue sections using the Duolink II Detection Kit (Olink Bioscience, Uppsala, Sweden) following the manufacturer’s protocol using primary antibodies against HIF-1α (clone ESEE122) and VHL (52A11; Abcam, Cambridge, UK) both at 2 μg/mL. DAPI was used as a counterstain. Heterodimers of these proteins (visualized as bright fluorescent signals) were examined with a laser scanning confocal microscope (Leica Lasertechnik, North Ryde, NSW, Australia). The number of PLA signals per cell was counted by semi-automated image analysis using BlobFinder V 3.0.

### 4.8. Statistical Analysis

Statistical significance was assessed by Mann–Whitney *U*-test with *p* < 0.05 considered significant. One normal SPT pregnancy control per gel served as a reference and changes observed in SPT, IUGR, and PE samples presented as fold change compared to this reference sample.

Table outlines sense (S) and anti-sense (AS) primers used, annealing temperatures, Mg^2+^ concentrations, product lengths, and cycle numbers for PCR analysis of the genes indicated.

## Figures and Tables

**Table 1:** Clinical characteristics of the study groups.

Participant Details	SPT (*n* = 13)	IUGR (*n* = 16)	*p* Value	PE (*n* = 19)	*p* Value
Maternal age (years)	33.1 ± 3.7	31.5 ± 4.7	NS	31.11 ± 5.99	NS
Nulliparity–no. (%)	9 (69.2)	10 (62.5)	-	9 (47.5)	-
Gestational age at sampling/delivery (weeks)	31.97 ± 2.87	31.97 ± 2.873	NS	32.77 ± 2.113	NS
Smoking–no. (%)	0 (0.0)	1 (6.2)	-	1 (5.3)	-
Maximum systolic BP (mmHg) Mean ± SD	117.5 ± 9.744	121.7 ± 9.046	NS	160.5 ± 18.08 **	*p* < 0.0001
Maximum diastolic BP (mmHg) Mean ± SD	71.6 ± 4.274	75.88 ± 10.08	NS	96.11 ± 11.4 **	*p* < 0.0001
Maximum urine protein/creatinine ratio (mg/mmol)	*N*/A	N/A	-	315.6 ± 243	-
Birth weight (g)	1879 ± 542.1	1285 ± 422.1	*p* < 0.01	1695 ± 652.9	NS
Birth weight < 3rd centile for gestational age–no. (%)	0 (0.0)	16 (100.0)	-	4 (21)	-
Abnormal umbilical artery Doppler	0 (0.0)	16 (100.0)	-	4 (21)	-
Infant sex (male/female)	7/6	8/8	-	8/11	-
Delivery by CSno. (%)	12 (92)	16 (100)		19 (100)	

Data are presented as means ± SD. *p* values are given for significant differences in comparison with SPT pregnancies. NS denotes no significant difference (*p* > 0.05). In comparisons between IUGR and PE, only significant differences between these groups are indicated. ** *p* <0.01.

**Table 2:** PCR primers and conditions used for oxygen sensing/response genes.

Gene	Primers		[Mg2 + ] mM	Annealing Temp	Product Length	Cycle Number
sFlt-1	SAS	5′ gcaccttggttgtggctgact 3′ 5′ gagcccgggggtctcattatt 3′	1.25	60 °C	643 bp	25
HIF-1α	SAS	5′ cgttgtgagtggtattattcagcacg 3′ 5′ ggtcatcagtttctgtgtcgttgc 3′	1.5	63 °C	248 bp	31
HIF-2α	SAS	5′ gctcctctcctcagtttgctc 3′ 5′ ctgttagctccacctgtgtaagtc 3′	1.5	63 °C	179 bp	30
β-actin	SAS	5′ gaggcccagagcaagagag 3′ 5′ ccagaggcgtacagggatag 3′	1.5	57 °C	200 bp	20

## Data Availability

Not applicable.
